# Vestibular deficit and recovery in rare forms of benign paroxysmal positional vertigo with downbeat nystagmus

**DOI:** 10.1007/s00405-026-10289-7

**Published:** 2026-05-21

**Authors:** Gorkem Ertugrul, Luigi Califano, Pasquale Viola, Maria Grazia Melillo, Alessia Astorina, Claudio Petrolo, Carla Laria, Anna Rita Fetoni, Giuseppe Chiarella

**Affiliations:** 1 Department of Audiology, Faculty of Health Sciences, Hacettepe University, Ankara, Turkey; 2 Postdoc Fellowship at Department of Audiology and Phoniatrics, San Pio Hospital, Benevento, Italy; 3 Department of Audiology and Phoniatrics, San Pio Hospital, Benevento, Italy; 4 Department of Experimental and Clinical Medicine, Unit of Audiology, Regional Center for Cochlear Implants and ENT Diseases, Magna Graecia University, Catanzaro, Italy; 5 Department of Audiology and Phoniatrics, University of Naples Federico II, Naples, Italy

**Keywords:** Benign paroxysmal positional vertigo, Downbeat nystagmus, Dizziness, Vestibular loss, Recovery

## Abstract

**Purpose:**

The primary goals are to investigate the rate of vestibular deficit (VD) during the acute attack and to follow up on vestibular recovery (VR) after the resolution of BPPV in patients with rare forms of BPPV that cause downbeat nystagmus. Second, to determine the effects of VD on dizziness severity in patients with rare BPPV forms and to compare all the results with those of typical posterior canal BPPV.

**Methods:**

This prospective follow-up study included 23 patients (56.86 ± 12.21 years) with rare BPPV forms (5 anterior canal, 18 apogeotropic posterior canal) and 23 patients (50.75 ± 13.67 years) with typical posterior canal BPPV. Dix-Hallpike, McClure-Pagnini, Semont, and supine head-hanging diagnostic maneuvers were performed on all patients during the acute BPPV attack. All patients underwent an objective (vHIT and oVEMP) and subjective vestibular test (DHI) battery before the therapeutic maneuvers (Epley, Quick Liberatory Rotation, or Yacovino maneuvers). One month after complete resolution of BPPV, all vestibular evaluations were performed once again in both groups.

**Results:**

In patients with rare forms of BPPV, 30.43% had canal deficit and 43.38% had utricular deficit on the affected side during an acute BPPV attack (p = 0.009, p = 0.001), whereas no patients in the typical BPPV group had vestibular deficit (0%). One month after BPPV resolution, the canal deficit of 26.09% and the utricular deficit of 34.78% had completely recovered in the rare forms of BPPV group. The rare BPPV forms group reported experiencing a higher level of dizziness during the acute attack and more residual dizziness one month after resolution, compared to the typical BPPV group, despite the fact that there are no statistically significant differences in dizziness levels between the groups (p > 0.05).

**Conclusion:**

It is possible that a VD may be seen in some patients with rare forms of BPPV and may not fully recover. It should be kept in mind that rare forms of BPPV may cause persistent VD in some patients, even if BPPV is resolved. The patients with both rare forms of BPPV (much more) and typical BPPV forms may describe residual dizziness after the resolution of BPPV. Additionally, canal deficit is a significant predictor of dizziness severity in all patients with BPPV.

**Graphical Abstract:**

**Figure:**
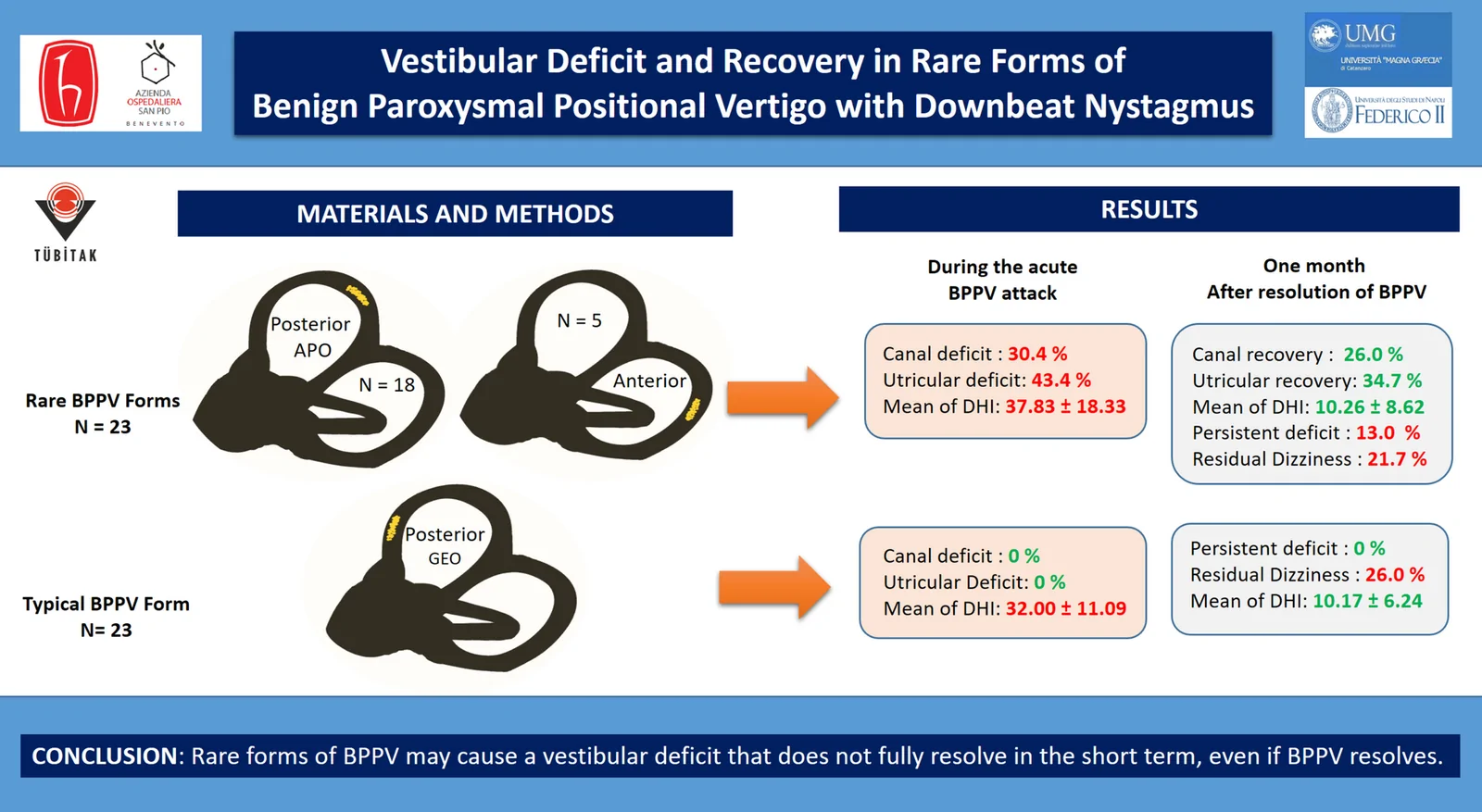

## Introduction

Spontaneous DBN, frequently considered a sign of central pathology, can also occur in peripheral vestibular disorders such as BPPV. In a study investigating potential causes of DBN in 54 patients with vertigo or dizziness, 51.9% had an identifiable etiology, with peripheral disorders accounting for 22.2% and central lesions for 29.6%. Vestibular migraine (13.0%) and cerebellar lesions (13.0%) were the most common underlying conditions, followed by BPPV (7.4%) [1].

BPPV is the most common peripheral vestibular disorder, with a lifetime prevalence of 2.4%, affecting 3.2% of females and 1.6% of males [2]. Canalithiasis and cupulolithiasis are the primary mechanisms explaining BPPV pathophysiology [3]. Typical PC-BPPV, characterized by otoconial debris in the ampullary side of the PC, leading to upbeating torsional nystagmus toward the affected side with brief latency, accounts for 80% of BPPV cases. The rare vertical canal forms of BPPV (5%) include anterior canal (AC) canalithiasis and apogeotropic posterior canal (APC) canalithiasis [4, 5].

This study focused on these two rare forms of BPPV, which mimic central vestibular disorders. These forms are characterized by peripheral DBN and positional vertigo triggered by head movements. Unlike typical PC-BPPV, APC-BPPV canalithiasis involves otoconial debris in the non-ampullary side of the PC, leading to positional DBN. The clinical characteristics of AC-BPPV and APC-BPPV are similar; however, the torsional component is not always distinct in AC-BPPV, making differentiation from central positional DBN challenging [6, 7].

There are limited studies on the differential diagnosis of these rare vertical canal forms of BPPV [1, 6, 8–11], and no comprehensive follow-up studies have investigated functional deficits in the affected utricle and semicircular canal (SCC) or recovery in these patients. The impact of vestibular loss on dizziness severity in these atypical BPPV forms remains unclear.

The primary goals are to investigate the rate of vestibular deficit (VD) during the acute attack and to follow up on vestibular recovery (VR) after the resolution of BPPV in patients with rare forms of BPPV that cause downbeat nystagmus. Second, to determine the effects of VD on dizziness severity in patients with rare BPPV forms and to compare all the results with those of typical posterior canal BPPV.

## Materials and methods

### Participants

This prospective follow-up study included a cohort of 23 patients (mean age: 56.86 ± 12.21 years, range: 29–73, female/male ratio = 0.91) diagnosed with rare forms of BPPV (study group), specifically AC- or APC-BPPV. A control group of 23 patients (mean age: 50.75 ± 13.67 years, range: 23–74, female/male ratio = 2.28) was also included, all diagnosed with typical PC-BPPV (control group). The PC was the most frequently affected canal in both BPPV groups (Table 1). APC-BPPV, which affected 18 patients, accounted for 43.4% (18/41) of all acute PC-BPPV cases and was significantly more prevalent than AC-BPPV (5 patients) in this study sample (APC/AC ratio: 3.6:1).

**Table 1:** Descriptive Statistics

		Group				
		Rare forms of BPPV			Typical PC BPPV	
			n	%	n	%
Gender	Male		12	52.2	7	30.4
	Female		11	47.8	16	69.6
	Total		23	100	23	100
Affected Canal	Anterior		5	21.7	-	-
	Posterior		18	78.3	23	100
	Total		23	100	23	100
Dizziness Severity During the Acute Attack (DHI Total)	No Handicap		1	4.3	2	8.7
	Mild Handicap		10	43.5	10	43.5
	Moderate Handicap		7	30.4	11	47.8
	Severe Handicap		5	21.7	0	-
	Total		23	100	23	100
Dizziness Severity 1 Month After the BPPV Resolution (DHI Total)	No Handicap		18	78.3	17	73.9
	Mild Handicap		5	21.7	6	26.1
	Moderate Handicap		0	-	0	-
	Severe Handicap		0	-	0	-
	Total		23	100	23	100

DHI: Dizziness Handicap Inventory; PC: Posterior Canal

Exclusion criteria were the presence of cognitive or neurological disorders such as dementia, balance disorders secondary to neurological conditions, Ménière’s disease, vestibular neuritis, or vestibular migraine. Participants using vestibular suppressant drugs, reporting hearing loss, or having age-related vestibular degeneration were also excluded. Individuals with severe motion limitations, lumbar or cervical disc herniation, or other musculoskeletal restrictions preventing positional maneuvers were not included in the study.

According to BPPV grading system for rare forms [7], 16 of the 18 patients with APC-BPPV were classified as having "definite" APC-BPPV, while the remaining 2 patients were classified as "probable" APC-BPPV. Conversely, 4 of the 5 patients with AC-BPPV were classified as having "definite" AC-BPPV, and 1 patient was classified as "probable" AC-BPPV [7].

### Study design

All data were collected at San Pio Hospital, Department of Audiology and Phoniatrics in Italy. A comprehensive vestibular evaluation was conducted for all patients. The diagnosis of BPPV was made using the Dix-Hallpike, McClure-Pagnini (supine roll test), side-lying test, and supine head-hanging diagnostic maneuvers [12, 13]. All positional nystagmus was recorded using videonystagmography with the Video Frenzel (*Nystalyze VNG module, Inventis, Padova, Italy*). The severity of dizziness was assessed using the Italian version of the DHI, and DHI scores were classified as follows: 0–30: mild handicap, 31–60: moderate handicap, 61 + : severe handicap [14, 15]. While the canal deficit and vestibulo-ocular reflex (VOR) gain of six canals were measured with the video Head Impulse Test (vHIT, *ICS Impulse Inventis, Padova, Italy*). Utricular deficit was evaluated using ocular Vestibular Evoked Myogenic Potentials (oVEMP, *Socrates-Hedera Inventis, Padova, Italy*) with 500 Hz tone-burst air-conduction stimuli, and N1-P2 waves were recorded at 120 dB SPL. In this study, “persistent vestibular deficit” was defined as the failure of utricular and/or canal function to improve despite resolution of BPPV.

Canal function was considered as “canal deficient” if the angular VOR gain was below 0.80 and corrective saccades (both overt and covert) were present in the affected canal [16, 17]. Utricular function was deemed as “utricular deficient” if the asymmetry rate exceeded 35% or if the oVEMPs response was absent on the affected side [18]. All patients underwent an objective (vHIT and oVEMP) and subjective vestibular test (DHI) battery before the therapeutic maneuvers. In the rare BPPV group, the Head-Shaking test and mastoid vibration (*Inventis Synapsis Vestibular Vibrator, Padova, Italy*) test (100 Hz vibration applied to the mastoid of the affected side for 1 min) were employed to convert AC and APC-BPPV forms into typical geotropic PC-BPPV [6, 7, 19]. After these assessments, repositioning maneuvers were performed as therapeutic interventions: the Epley [20–22] or Quick Liberatory Rotation (QLR) maneuver [23] for treating typical PC-BPPV and APC-BPPV, and the Yacovino maneuver [24] for treating AC-BPPV. A forced prolonged position was prescribed as a home procedure [25]. Resolution of BPPV was defined as the absence of elicited positional nystagmus in this study. In the typical PC-BPPV group, BPPV was resolved in all patients during the first session. However, in the group of rare BPPV variants, 3 patients diagnosed with probable BPPV grade (2 of them APC, 1 of them AC) did not exhibit conversion to the typical posterior canal form during therapeutic maneuvers. In these cases, BPPV was resolved by the second follow-up session (one week later). One month after complete resolution of BPPV, all vestibular evaluations were performed once again in both groups.

### Statistical analyses

The Kolmogorov–Smirnov test confirmed that DHI scores were normally distributed (p > 0.05). Independent sample t-tests were used to compare differences between the total DHI scores of both groups. Paired sample t-tests were applied for within-group comparisons of the total DHI scores during the acute BPPV episode and 1 month after BPPV resolution. Multiple regression analysis (enter and stepwise methods) was conducted to investigate the influence of VD on the severity of functional dizziness. VOR gain (canal function) and P1-N1 response (utricular function) were categorized as 1 = Deficit and 0 = No deficit. A p-value of < 0.05 was considered statistically significant. All statistical analyses were performed using IBM SPSS Statistics 25.0 software (SPSS, 2017).

## Results

**Fig. 1:**
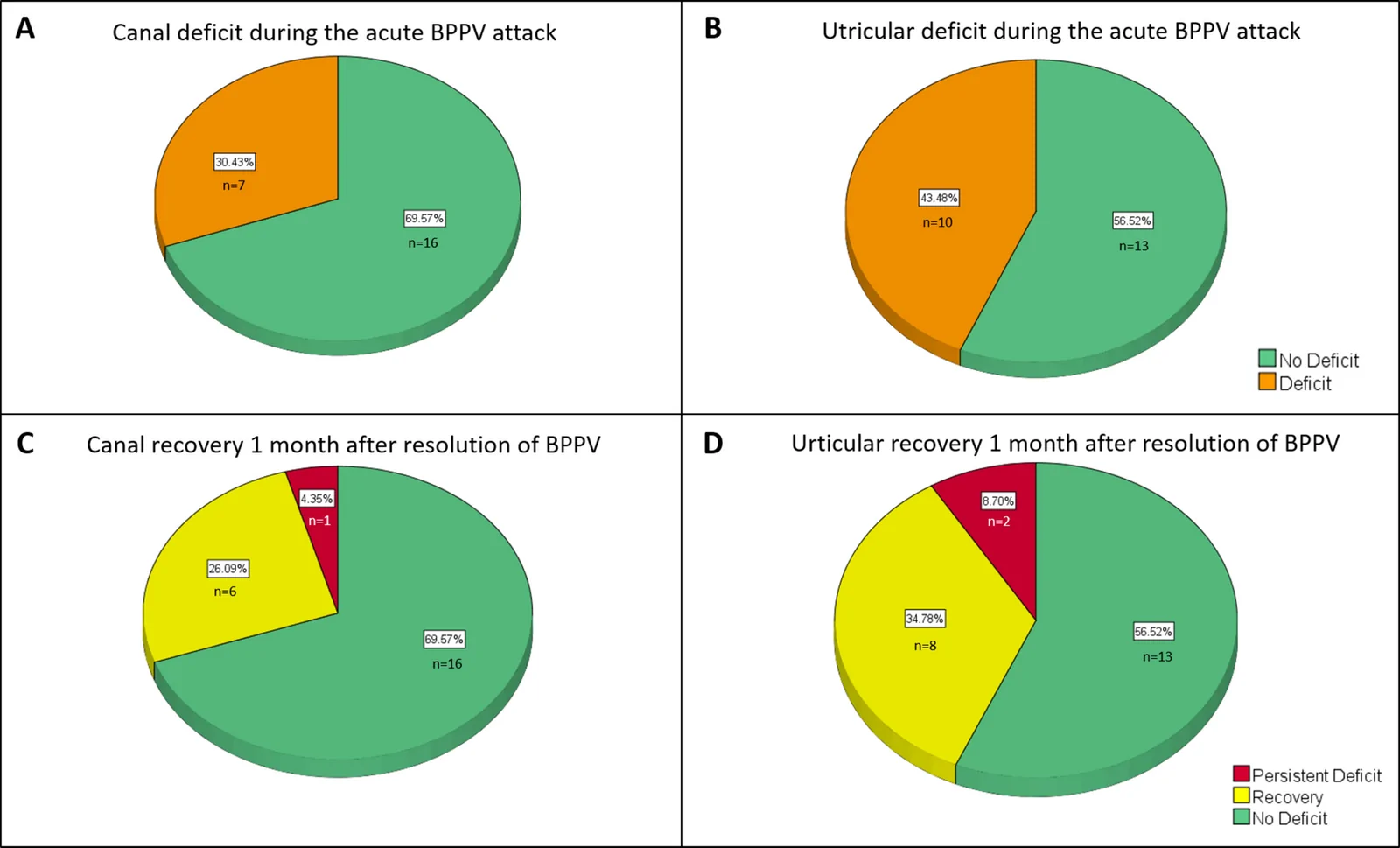
Objective indicators of vestibular deficit and recovery in rare forms of BPPV. (**A)** Canal deficit and **(B)** Utricular deficit during the acute BPPV attack. (**C)** Canal recovery and (**D)** Utricular recovery 1 month after resolution of BPPV. There was no vestibular deficit in the typical PC BPPV group

Consistent with the study by Califano et al. Califano, Mazzone [7] (6 times), APC-BPPV was more common (3.6 times) than AC-BPPV in this research, even with the small sample size. During the acute BPPV attack phase, a significant difference was observed between the rare BPPV group (mean VOR gain of the affected canal = 0.74 ± 0.088) and the typical PC-BPPV group (mean VOR gain of the affected canal = 0.87 ± 0.08) concerning the VOR gain of the affected canal (t_(44)_ = −3.636; p = 0.001). In the rare BPPV group, during the acute BPPV attacks, 7 patients (30.43%) exhibited canal deficits (2 with AC-BPPV, 5 with APC-BPPV) on the affected canal, and 10 patients (43.48%, 2 with AC-BPPV and 8 with APC-BPPV) showed utricular deficits (7 with absent P1-N1 waves, 3 with decreased P1-N1 amplitude) on the affected side (Fig. 1A and B). Significant differences were found between the rates of canal and utricular deficits on the affected side in the rare BPPV group compared to the typical PC-BPPV group (Fisher’s Exact Test, p = 0.009, p = 0.001, respectively).

As a primary outcome of the current study, one month after the resolution of BPPV, in the rare BPPV group, 6 patients (26.09%) had complete recovery of the canal deficit, while 1 patient (4.35%) exhibited a persistent canal deficit (no recovery in mean VOR gain) despite resolution of APC-BPPV. Furthermore, as shown in Fig. 1C-D, 8 patients (34.78%) had complete recovery of the utricular deficit, while 2 patients (1 with AC-BPPV and 1 with APC-BPPV) (8.70%) demonstrated persistent utricular deficits (still absent P1-N1). In the control group, all patients with typical PC-BPPV showed no canal deficit in the affected canal (mean VOR gain > 0.80 for each patient) and no utricular deficit on the oVEMPs test (all P1-N1 waves were present and symmetrical at 120 dB SPL on both sides for each patient).

**Fig. 2:**
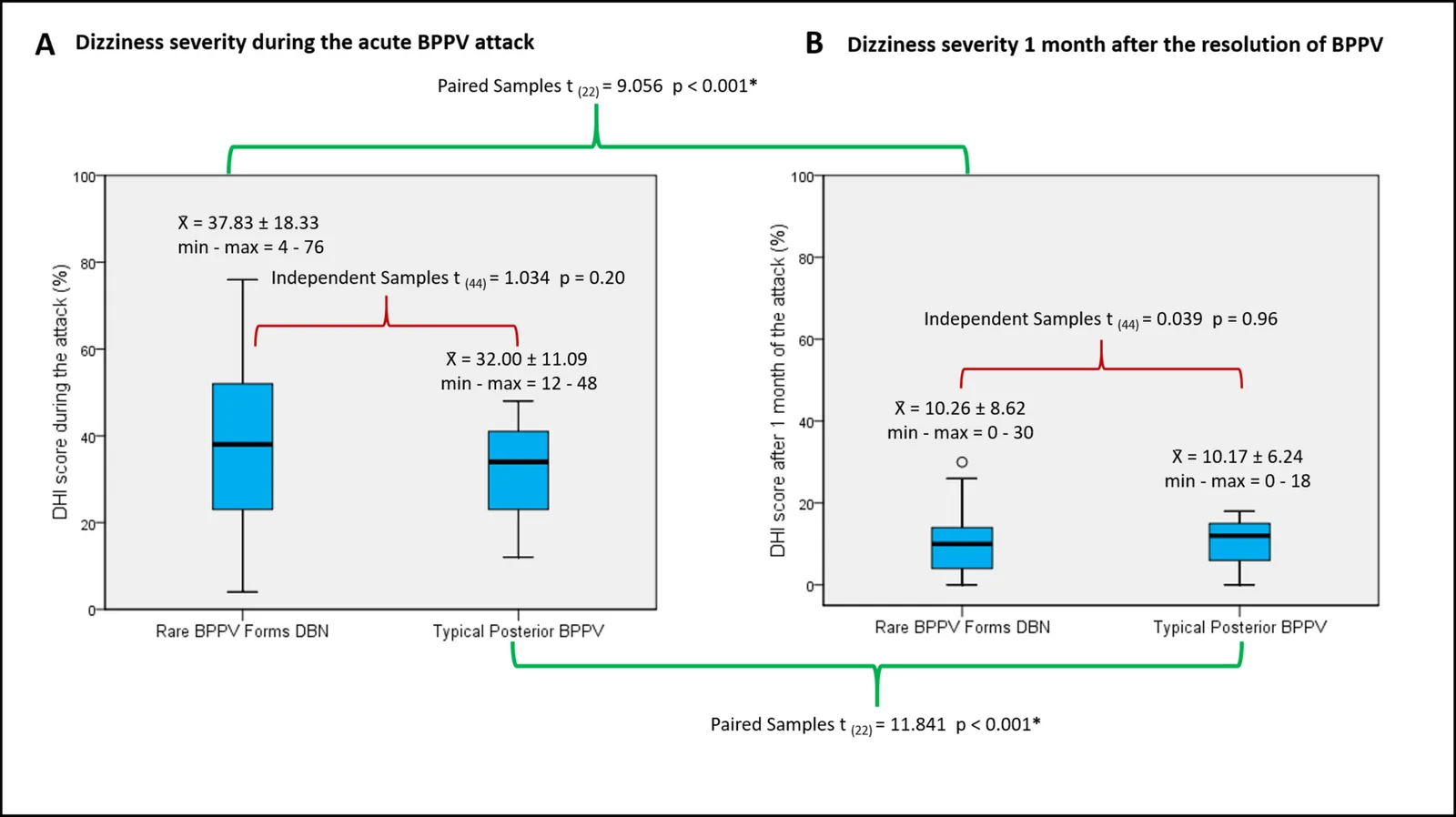
Subjective indicators of vestibular deficit and recovery in both groups. **(A)** Dizziness severity during the acute BPPV attack. **(B)** Dizziness severity 1 month after the resolution of BPPV

Secondly, during the acute BPPV attack, 5 of 23 patients (21.7%) with rare BPPV forms experienced severe dizziness handicap, whereas none of the patients in the typical PC-BPPV group had severe dizziness handicap (Table 1). Between-group comparisons of total DHI scores (Fig. 2, red lines) revealed no statistically significant difference in dizziness severity between the rare BPPV and typical PC-BPPV groups (p > 0.05). However, during the acute BPPV attack, the mean DHI physical subscale scores were significantly higher in the rare BPPV group compared to the typical PC-BPPV group (mean DHI physical subscale = 17.48 ± 5.632 in the rare BPPV group, mean DHI physical subscale = 14.30 ± 4.646 in the typical PC-BPPV group; independent sample t-test t_(44)_ = 2.085, p = 0.043).

As shown in Fig. 2 (green lines) and Table 2, within-group comparisons for both the rare BPPV and typical PC-BPPV groups demonstrated significant differences in total DHI scores and DHI subscale scores between the acute BPPV attack and 1 month after BPPV resolution (paired sample t-test p < 0.001). DHI scores significantly decreased in both groups 1 month after the resolution of BPPV. However, 11 of 46 patients (23.9%) with BPPV (5 with rare forms and 6 with typical PC-BPPV) continued to experience mild residual dizziness 1 month after BPPV resolution (Table 1).

**Table 2:** Comparisons of within-group in terms of dizziness severity during the acute BPPV attack and one month after the resolution of BPPV

Group	DHI score	During the acute BPPV attack	1 month after the resolution of BPPV	p value
		mean ± SD	mean ± SD	
Rare BPPV	Total	37.83 ± 18.33	10.26 ± 8.62	< 0.001*
	Functional	14.00 ± 8.75	3.48 ± 3.57	< 0.001*
	Emotional	6.35 ± 6.28	1.78 ± 3.30	< 0.001*
	Physical	17.48 ± 5.63	5.00 ± 3.98	< 0.001*
Typical PC BPPV	Total	32.00 ± 11.09	10.17 ± 6.24	< 0.001*
	Functional	11.83 ± 6.05	3.57 ± 2.95	< 0.001*
	Emotional	5.87 ± 4.35	2.09 ± 2.37	< 0.001*
	Physical	14.30 ± 4.64	4.52 ± 3.77	< 0.001*

PC: Posterior canal; DHI: Dizziness Handicap Inventory; BPPV: Benign Paroxysmal Positional Vertigo; * paired sample t test statistically accepted significance level p < 0.05

Additionally, a multiple linear regression analysis was performed to predict dizziness severity based on canal function (VOR gain by vHIT) and utricular function (oVEMPs). A significant regression equation was found using the stepwise method (F _(1,44)_ = 8.752; p = 0.005), with an adjusted R^2^ of 0.147. Canal function (VOR gain) and utricular function (P1-N1 response) were coded as 1 = Deficit and 0 = No deficit. The canal function was found to be a significant predictor of dizziness severity. During the acute BPPV attacks, for each 1-unit decrease in VOR gain, the DHI score increased by 17.121 (a negative correlation r = −0.407). In other words, canal function accounted for 14.7% of the variance in the DHI scores (Table 3).

**Table 3:** Multiple Regression Results

	β^1^ (%95 CI)	SE	β^2^	t	p	Correlation (Zero-order)	VIF
Method (Enter)							
(Constant)	51.666 (40.505—62.827)	5.534	0.000	9.335	0.000		
Canal Function	−12.532 (−25.979—0.916)	6.668	−0.298	−1.879	0.067	−0.407	1.352
Utricular Function	−7.830 (−19.541- 3.880)	5.807	−0.214	−1.349	0.185	−0.366	1.352
Method (Stepwise)^a^							
(Constant)	40.429 (38.689—60.168)	5.329	0.000	9.276	0.000*		
Canal Function	−17.121 (−28.784 −5.457)	5.787	−0.407	−2.958	0.005*	−0.407	1.000

β^1^: Unstandardized Coefficients; β^2^: Standardized Coefficients; Dependent Variable: Dizziness Handicap Inventory Score * F _(1,44)_ = 8.752; p = 0.005; R^2^ = 0.166; Adjusted R^2^ = 0.147; SE of Estimate = 14.099; Durbin-Watson = 2.154; ^a^ Predictors: (Constant), Canal function 0 = No Deficit; Excluded Variables: Utricular function = No deficit p = 0.185; * accepted significance level p < 0.05

The results of this study are illustrated in the graphical abstract.

## Discussion

This study represents a comprehensive investigation into functional VD and VR in patients with rare forms of BPPV leading to positional DBN, compared to typical PC-BPPV. Additionally, the impact of VD on dizziness severity during acute BPPV attacks was assessed.

Objective vestibular tests play a crucial role in the differential diagnosis of nystagmus, particularly in distinguishing between peripheral and central origins. Videonystagmography (VNG) and/or video Frenzel goggles are commonly employed to visualize and record positional nystagmus during diagnostic maneuvers and to monitor changes during therapeutic procedures. However, VNG and video Frenzel goggles alone are insufficient to differentiate peripheral DBN, particularly in cases of AC and APC-BPPV, as these forms can present similarly to central positional nystagmus. The vHIT offers an objective method to evaluate each SCC individually. Califano, Iannella [10] suggest that vHIT is not essential for diagnosing the typical geotropic form of PC-BPPV, while Castellucci, Malara [8] recommend vHIT for distinguishing rare forms of AC and APC-BPPV, showing a sensitivity of 88.6%. Previous studies have reported lower VOR gains and corrective saccades in the affected canal during the acute phase of rare canalithiasis forms of BPPV, causing DBN [1, 9]. Zhang, Lang [1] demonstrated that PC VOR gains were lower than AC VOR gains in BPPV cases, causing DBN, depending on the affected canal. In this study, the rate of canal deficit in APC-BPPV patients (5/18) was higher compared to AC-BPPV patients (2/5), consistent with previous findings. Moreover, in a rare case report of APC-BPPV associated with SCC dehiscence, fluctuations in high-frequency VOR gain were observed before and after therapeutic maneuvers [11]. Previous literature consistently reports that VOR gain and SCC function are typically normal in patients with typical PC-BPPV [10, 26, 27]. In line with these findings, our study showed that while canal deficit was observed in 30.43% of patients with rare BPPV forms during the acute BPPV attack, all patients with typical PC-BPPV had normal canal function (VOR gains within the normal range). Additionally, as reported by Castellucci, Malara [9], canal recovery was observed in 26.03% of patients with rare BPPV forms who had canal deficits during the acute attack, one month after BPPV resolution. However, one patient (4.36%) exhibited persistent canal deficit despite resolution of APC-BPPV, which is termed as persistent canal deficit.

The vHIT test, while informative for assessing SCC function, does not provide insights into utricular function, another crucial vestibular structure in BPPV. In addition to vHIT, oVEMP evaluating utricular function is a valid objective indicator to measure the effect of therapeutic maneuvers in BPPV [28]. Manzari et al. [27] reported that in 88.8% of cases, acute PC-BPPV patients exhibited intact SCC and utricular function during the acute phase, which aligns with the findings in the typical PC-BPPV group of this study. Previous studies have shown that utricular function is more frequently affected than saccular function in BPPV patients [29–35]. In our study, the number of patients with utricular deficit was greater in APC-BPPV (8/18) compared to AC-BPPV (2/5). Utricular dysfunction during acute BPPV may be attributed to the recurrence of BPPV episodes, as suggested by prior research [31, 32, 36]. A systematic review reported recurrence rates of BPPV ranging from 10 to 18% [32], while Mendeš, Maslovara [37] indicated that 18.75% of patients experienced recurrence, with oVEMPs asymmetry ratio (AR) being a strong predictor of recurrence. However, as a limitation, our study did not monitor BPPV recurrence during the data collection period. Regardless of recurrence, our findings show that 43.48% of patients with rare BPPV forms had utricular deficits during the acute attack, while all patients in the typical PC-BPPV group exhibited normal utricular function. Notably, two patients (8.70%) with rare BPPV variants demonstrated no recovery of utricular function one month following BPPV resolution. Among these, one patient with AC-BPPV exhibited a persistent utricular deficit [38], despite experiencing only a mild dizziness handicap both before and after BPPV treatment (pre-DHI total: 20; post-DHI total: 6). Another patient with APC-BPPV presented with both persistent utricular and canal deficits, referred to as persistent VD. This patient experienced a moderate dizziness handicap before BPPV treatment (pre-DHI total: 40), although the dizziness level markedly decreased to a mild dizziness handicap after BPPV resolution (post-DHI total: 2).

This study also supports findings in the literature suggesting that a retesting interval of one week or less is insufficient to detect VD complete recovery resulting from an acute BPPV episode, particularly concerning utricular macula function. In contrast, our study assessed VR one-month post-resolution, which provided more comprehensive insights into recovery patterns. Mendeš, Maslovara [37] observed significant asymmetry in oVEMPs readings seven days post-Epley maneuver, with statistically significant changes occurring only after six months. This underscores the importance of extending follow-up periods for assessing VR. On the other hand, based on objective findings in the current research, it is possible to conclude that VD resulting from rare BPPV forms completely recovered following the resolution of BPPV. It may indirectly demonstrate that a previous VD in these patients, regardless of BPPV, was ruled out.

The DHI is a widely used subjective assessment tool for evaluating dizziness severity in BPPV patients [39]. A correct BPPV diagnosis is significantly more likely with a DHI score of 50 or higher [40]. While previous studies have largely focused on dizziness severity in typical PC or other canal BPPV forms [39–41], few have addressed dizziness severity in rare BPPV variants such as APC and AC-BPPV. In this study, although there was no statistically significant difference in dizziness severity between patients with rare forms of BPPV and typical PC-BPPV, patients with rare forms of BPPV reported higher dizziness severity during the acute phase. Interestingly, the severity of dizziness (including its physical, emotional, and functional aspects) decreased significantly one month after BPPV resolution in both groups.

Residual dizziness is a known risk factor for post-treatment complications in BPPV [42–48]. In this study, 23.9% of all BPPV patients experienced residual vertigo after the resolution of BPPV. This indicates that some individuals continue to experience mild vertigo even after recovery. Martellucci et al. [49], in their study examining the determinants of residual dizziness following BPPV treatment, found that resumption of daily life activities significantly reduced the likelihood of experiencing residual dizziness by approximately 14 times, regardless of patients’ age, sex, or the number of repositioning maneuvers performed. As another limitation of this study, due to the limited sample size of rare BPPV forms, patients' daily life activities were not controlled. Additionally, based on the regression analysis results of our study, it is possible to state that canal deficit is a significant determinant of dizziness severity in patients with BPPV. However, it is recommended that these results be replicated with a larger sample size of rare BPPV forms.

In conclusion, this study demonstrates that rare forms of BPPV, which are frequently mistaken for central pathologies, can lead to persistent vestibular deficits in some patients. Future research should focus on larger sample sizes to further elucidate the distinct characteristics of AC and APC-BPPV and investigate these forms in separate groups to enhance clinical understanding and diagnostic accuracy.

## Conclusion

It is possible that a VD may be seen in some patients with rare forms of BPPV and may not fully recover. It should be kept in mind that rare forms of BPPV may cause persistent VD in some patients, even if BPPV is resolved. The patients with both rare forms of BPPV (much more) and typical BPPV forms may describe residual dizziness after the resolution of BPPV. Additionally, canal deficit is a significant predictor of dizziness severity in all patients with BPPV.
